# Hydrogel Surface/Coating for Smart Drug Delivery and Medical Devices

**DOI:** 10.3390/gels10090592

**Published:** 2024-09-13

**Authors:** Changcan Shi, Xingzhou Peng, Yakai Feng

**Affiliations:** 1 Wenzhou Institute, University of Chinese Academy of Sciences, Wenzhou 325011, China; shicc@wibe.ac.cn; 2 State Key Laboratory of Digital Medical Engineering, Key Laboratory of Biomedical Engineering of Hainan Province, School of Biomedical Engineering, Hainan University, Sanya 572025, China; pengxzh@hainanu.edu.cn; 3 School of Chemical Engineering and Technology, Tianjin University, Tianjin 300350, China; 4 Frontiers Science Center for Synthetic Biology, Tianjin University, Tianjin 300072, China; 5 Key Laboratory of Systems Bioengineering (Ministry of Education), Tianjin University, Tianjin 300072, China

In recent years, multifunctional hydrogels have been used to develop emerging medical platforms and become an alternative approach in targeting therapies and tissue regeneration. Hydrogels have unique soft, gentle, and responsive characteristics, and endow them with huge potential in medical applications, such as artificial organs, biomedical sensors, drug/gene delivery, and the surface modification of medical devices. In particular, hydrogel surface and coatings have recently been explored as smart drug delivery systems and used to modify medical devices. Thus, this Research Topic focuses on recent advances in hydrogel surface/coatings for smart drug delivery and medical devices.

This Research Topic is covered by five articles, including three original research articles and two review articles. The original research articles involved smart temporary microgel films in delivery drugs for treating cancers [1], poly (aldehyde guluronate) hydrogels for the controlled delivery of growth factors and promoting vascularization in regenerative medicine [2], and semi-liquid 2.5% polyacrylamide hydrogel as a scaffold for fluorescent polyelectrolyte microcapsules in rainbow trout [3]. These hydrogels in the three original research articles were rationally designed and demonstrated efficient results. There are two review articles on this Research Topic. One review paper systematically introduced the main synthetic strategies for creating lubricating polymer gels/coatings at the molecular level and measurements of the reduced-friction materials [4]. The other review paper introduced chitosan hydrogels, and their modification and application in tissue engineering scaffolds for vascular regeneration [5].

In summary, the current Research Topic reports the recent significant advances in hydrogel surface/coatings for smart drug delivery and medical devices. The design and development of functional hydrogels will provide new opportunities for smart drug delivery towards clinical application and the development of medical devices. The articles in this Research Topic will be a helpful reference for hydrogels, surface modification, and drug/gene delivery.
